# *In Vivo *Transfer of Intracellular Labels from Locally Implanted Bone Marrow Stromal Cells to Resident Tissue Macrophages

**DOI:** 10.1371/journal.pone.0006712

**Published:** 2009-08-21

**Authors:** Edyta Pawelczyk, Elaine K. Jordan, Arun Balakumaran, Aneeka Chaudhry, Nicole Gormley, Melissa Smith, Bobbi K. Lewis, Richard Childs, Pamela G. Robey, Joseph A. Frank

**Affiliations:** 1 Radiology and Imaging Sciences, Clinical Center, National Institutes of Health, Bethesda, Maryland, United States of America; 2 National Institute of Dental and Craniofacial Research, National Institutes of Health, Bethesda, Maryland, United States of America; 3 National Heart, Lung and Blood Institute, National Institutes of Health, Bethesda, Maryland, United States of America; 4 National Institute of Biomedical Imaging and Bioengineering, National Institutes of Health, Bethesda, Maryland, United States of America; Julius-Maximilians-Universität Würzburg, Germany

## Abstract

Intracellular labels such as dextran coated superparamagnetic iron oxide nanoparticles (SPION), bromodeoxyuridine (BrdU) or green fluorescent protein (GFP) are frequently used to study the fate of transplanted cells by *in vivo* magnetic resonance imaging or fluorescent microscopy. Bystander uptake of labeled cells by resident tissue macrophages (TM) can confound the interpretation of the presence of intracellular labels especially during direct implantation of cells, which can result in more than 70% cell death. In this study we determined the percentages of TM that took up SPION, BrdU or GFP from labeled bone marrow stromal cells (BMSCs) that were placed into areas of angiogenesis and inflammation in a mouse model known as Matrigel™ plaque perfusion assay. Cells recovered from digested plaques at various time points were analyzed by fluorescence microscopy and flow cytometry. The analysis of harvested plaques revealed 5% of BrdU^+^, 5–10% of GFP^+^ and 5–15% of dextran^+^ macrophages. The transfer of the label was not dependent on cell dose or viability. Collectively, this study suggests that care should be taken to validate donor origin of cells using an independent marker by histology and to assess transplanted cells for TM markers prior to drawing conclusions about the *in vivo* behavior of transplanted cells.

## Introduction

During the past few years, stem cell therapy has emerged as a promising alternative for treatment of various incurable diseases. Among many stem cell types, human bone marrow stromal cells (BMSCs) appear to hold a great advantage because they are easily obtained, propagated in culture and maintain genetic stability. BMSCs also have the potential for multi lineage differentiation to make bone, marrow adipocytes and hematopoietic supporting stroma. [1]. BMSCs can down-regulate several functions of immune cells, in addition to promoting survival of damaged cells and tissues through paracrine factors, possibly under the guidance of environmental cues [2]–[4]. BMSCs can also serve as cellular vehicles for the delivery of therapeutic proteins based on their ability to preferentially home to injured and inflamed tissues [5]–[7]. Thus, BMSCs clinical applications supported by preclinical results in different animal models are becoming a reality.

BMSCs are usually delivered either by intravenous injection or by direct implantation into target tissue. To track the fate of these cells in both clinical studies and animal models, cell labeling techniques such as bromodeoxyuridine (BrdU), green fluorescent protein (GFP) or superparamagnetic iron oxide nanoparticle (SPION) labeling, are being developed for tracking implanted cells on histological examimation, or *in vivo* visualization by noninvasive magnetic resonance imaging (MRI).

BrdU is a thymidine analogue that incorporates into DNA of dividing cells during the S phase of the cell cycle [8]. It has been widely used in various animal models to track migration and differentiation of transplanted cells by autopsy and two-photon microscopy [9]–[13]. In particular, BrdU labeling is currently the most commonly used method for studying neurogenesis in adult brain [14]. The introduction of GFP has revolutionized the field of cell biology and fluorescence microscopy [15]. GFP is a naturally fluorescent protein, originally cloned from jellyfish, *Aequoria victoria* that can be expressed in any species, in which genetic manipulation is possible. It is extensively used in animal models, in transplantation studies to determine the fate of transplanted cells, as well as for studying various biological processes.

BMSCs have also been more recently labeled after complexing with two FDA approved agents, ferumoxides (Fe), a dextran-coated SPION and protamine sulfate (Pro) to facilitate monitoring by magnetic resonance imaging (MRI) or Prussian blue staining of tissues [16]–[19]. MRI-based tracking of cells labeled with ferumoxides allows for non-invasive *in vivo* detection and longitudinal assessment of implanted cells [20]. Fe-Pro labeled cells appear as hypointense regions on MR images and thus can be distinguished from the surrounding tissue.

A concern with the use of markers (BrdU, SPION or GFP) to label cells is that the tag may be transferred to local, endogenous bystander cells, such as tissue macrophages, especially in areas of damage and inflammation, potentially confounding the interpretation of microscopy or cellular imaging. This is of particular importance in direct implantation of BMSCs into target tissues, which can result in 50% to 80% cells undergoing cell death [21]–[23]. Multiple reports have cautioned against over-interpretation of results of labeled transplanted cells to host cells, though there has been a dearth of reports quantifying the actual number of activated tissue macrophages engulfing the markers or marked cells [24]–[29]. Recently, Pawelczyk et al [30] in an *in vitro* study showed BrdU or SPION transferred to 10–20% of activated macrophages. Whether similar results would occur *in vivo* is unknown.

Our objective was to determine the percentages of host tissue macrophages that took up cellular labels, BrdU, GFP and SPION from labeled BMSCs in an *in vivo* localized model of angiogenesis and inflammation. Quantifying the percentage of macrophages that incorporate GFP, SPION or BrdU from viable or dead cells will aid in the interpretation of transplantation studies with labeled cells.

## Results

### Lentiviral transduction of BMSCs and labeling with BrdU and superparamagnetic iron oxide nanoparticles

**Figure 1:**
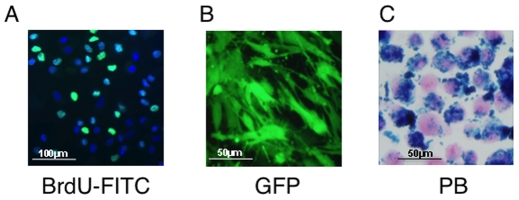
Labeling of human bone marrow stromal cells (BMSCs) with BrdU, GFP and Fe-Pro. Representative fluorescent microscopy images of BrdU – labeled hBMSCs stained with anti BrdU- FITC antibody (A), GFP expressing hBMSCs after lentiviral transduction (B) and Prussian Blue (PB) stained Fe-Pro labeled hBMSCs (C), magnification 40X.

Isolated human and mouse BMSCs were characterized, as previously published, by FACS, and for their potential for osteogenic, adipogenic, and chondrogenic differentiation in *in vitro* assays using specific induction media (data not shown) [1]. Lentiviral transduction resulted in ∼60% of hBMSCs expressing GFP. Only cell sorted GFP positive cells were used in animal experiments. Incubation of human or mouse BMSCs with BrdU resulted in ∼70% of cells being labeled as assessed by FACS. Prussian blue staining of Fe-Pro labeled mouse or human BMSCs revealed abundant uptake of the Fe-Pro complex into the cytoplasm in approximately 100% of cells. Representative microphotographs of Fe-Pro, GFP and BrdU labeled human BMSCs are presented in Figure 1. The iron content of Fe-Pro labeled BMSCs was 29.6±0.45 picogram per cell and was similar to the previously published studies [16].

### BrdU uptake by macrophages infiltrating Matrigel™ plaque

**Figure 2:**
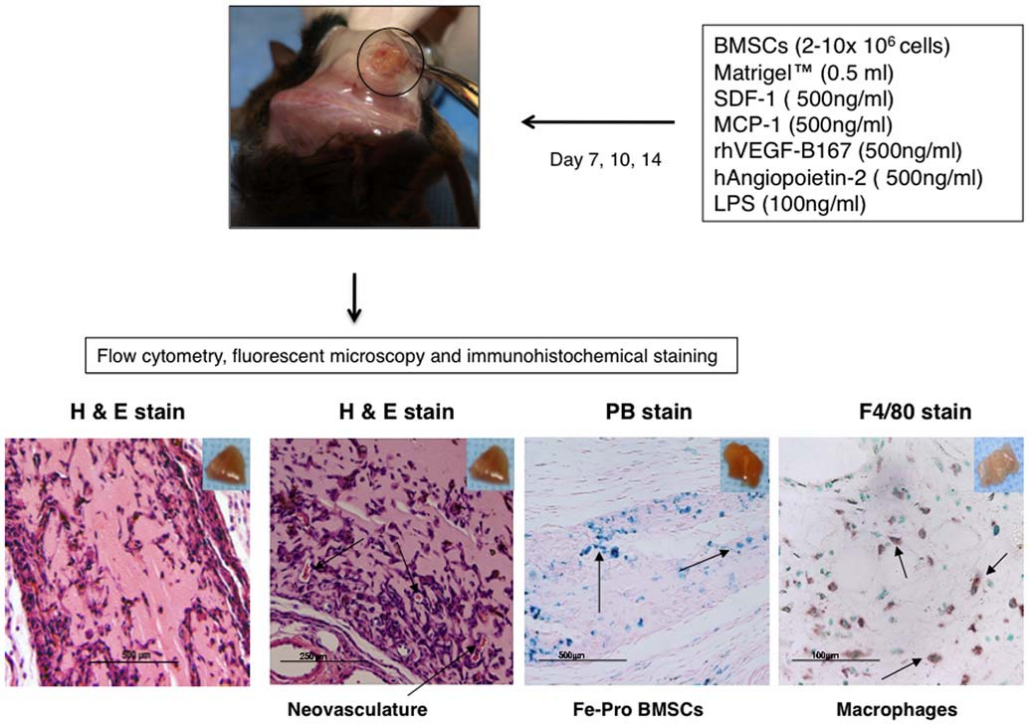
Schema of modified Matrigel™ plaque perfusion assay. Matrigel™ plaques (MPs) containing labeled bone marrow stromal cells (BMSCs), growth factors, chemokines and inflammatory molecules, are implanted into both flanks of 6-week-old 129/SvlmJ mice, and harvested at various time points for flow cytometry and microscopy analysis. Note representative images of collected MPs and immunohistochemical stains revealing neovasculature on H & E staining, iron-labeled cells on Prussian blue (PB) staining or the presence of F4/80 positive macrophages (brown cells), magnification 20X.

**Figure 3:**
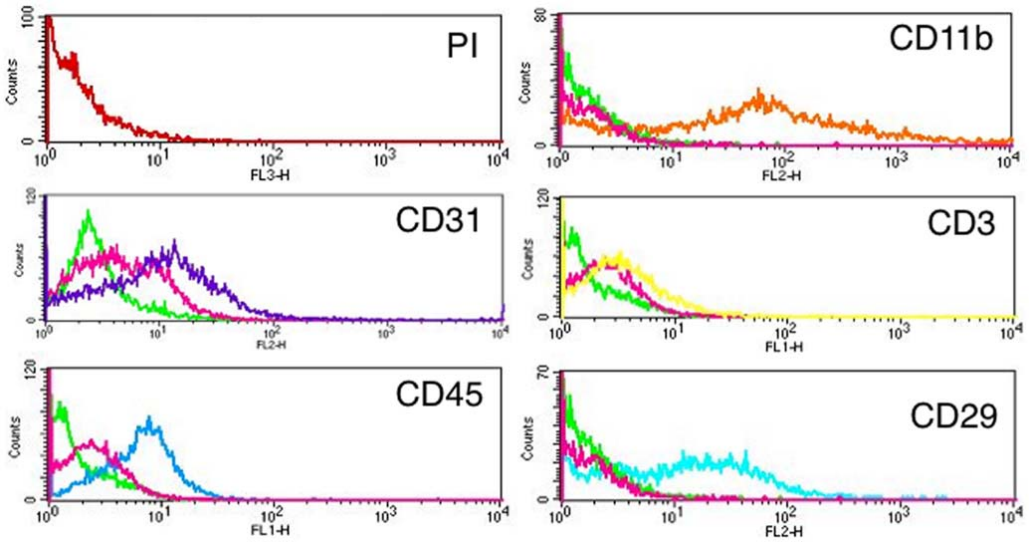
Matrigel™ plaque cell content harvested on day seven post-implantation. Flow cytometry histograms revealing live, propidium iodide (PI) negative cells expressing CD31, CD45, CD11b and CD3. Human BMSCs were detected by staining for CD29. Green lines indicate unstained cells, while pink lines cells stained with appropriate isotype antibodies.

**Figure 4:**
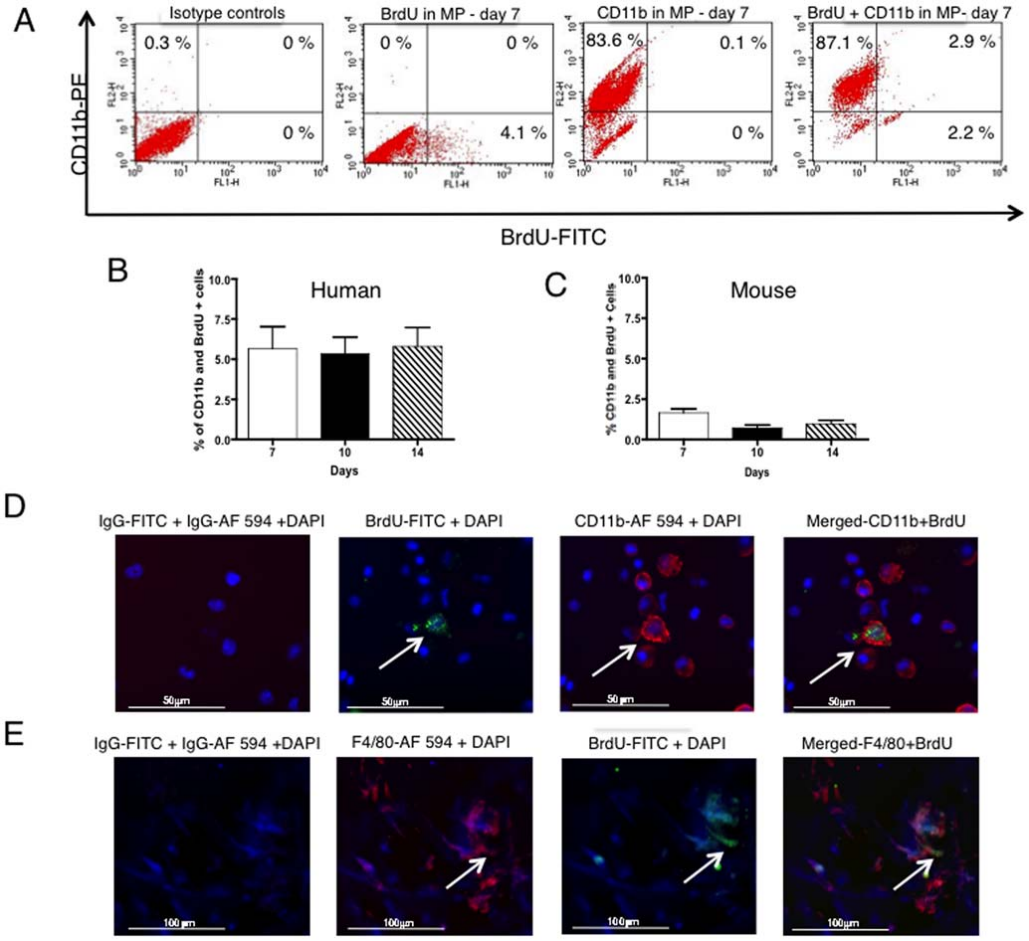
BrdU uptake by local infiltrating Matrigel™ plaque macrophages from BrdU-labeled BMSCs. Representative flow cytometry dot plots showing 2.9% of CD11b-PE and BrdU-FITC positive macrophages in MPs harvested seven days post-implantation (A), Bar graph showing the mean percentage of BrdU^+^ CD11b^+^ cells in all MPs collected 7, 10 and 14 days after implantation of MPs containing 2×10^6^ xenogenic hBMSCs (B) and 2×10^6^ syngeneic mouse BMSCs (C). Note the uptake of BrdU by tissue macrophages was lower from syngeneic BrdU – labeled mouse BMSCs when compared to xenogenic BrdU-labeled hBMSCs. Immunofluorescent detection of BrdU with anti-BrdU-FITC, CD11b with anti-mouse CD11b-Alexa Fluor 594 on cytospins of cells recovered from MPs seven days post-implantation (magnification 40X), (D). On the merged image, note cells only positive for CD11b-Alexa Fluor 594 and macrophages positive for BrdU-FITC and CD11b-Alexa Fluor 594. Those cells are local macrophages that took up BrdU from BrdU labeled hBMSCs. Immunofluorescent detection of BrdU with anti-BrdU-FITC, F4/80 with anti-mouse F4/80-Alexa Fluor 594 on frozen sections of MPs seven days post-implantation (magnification 40X), (D). Cells positive for macrophage marker, F4/80, as well as BrdU are indicated by arrows. Data are presented as a mean±1 SD from 12 plaques.

To determine how often exogenous intracellular labels from implanted BMSCs transfer or get taken up by infiltrating tissue macrophages in areas of inflammation, a modified Matrigel™ plaque perfusion assay was used (Figure 2). Matrigel™ plaques (MPs) were prepared by mixing Matrigel™ with a cocktail composed of growth factors, chemokines, LPS and labeled BMSCs, and then implanted subcutaneously into mice [31]–[33]. The number of live BMSCs implanted was chosen based on our and others previously published studies [30], [34]. Based on the hematoxylin and eosin (H& E) staining of the MPs sections, as early as day seven post-implantation, we observed new vessels being formed (Figure 2). To determine the type of cells migrating into the plaques, MPs harvested on day 7, 10 and 14 post-implantation were digested and analyzed by FACS and immuno-histochemistry techniques. The propidium iodide (PI) staining of MPs showed no dead cells were detected. The FACS analysis revealed cells expressing endothelial marker CD31, abundant presence of CD45^+^ positive cells including high levels of CD11b^+^ or F4/80^+^ cells and low numbers of CD3^+^ lymphocytes (Figure 2 and 3). Live and labeled human BMSCs that were originally implanted were detected based on CD29 staining (Figure 3). Flow cytometry analysis of cells recovered from digested plaques containing xenogeneic, human BrdU-labeled BMSCs showed that an average 5% of macrophages were CD11b^+^ BrdU^+^ (5.7%±3.03 on day 7, 5.4%±2.49 on day 10 and 5.8%±2.91 on day 14, p = not significant, between time points) (Figure 4A and 4B). When we analyzed the plaques containing BrdU- labeled syngeneic, mouse BMSCs, only 1.7%±0.68 of CD11b^+^ BrdU^+^ cells were found on day 7, 0.7%±0.49 on day 10 and 0.98%±0.58 (p = not significant, between time points) were CD11b^+^ BrdU^+^ two weeks after implantation (Figure 4C). The CD11b^+^ BrdU^+^ cells were visualized on cytospins and frozen sections under fluorescence microscope (Figure 4D and 4E). The percentage of CD11b^+^ BrdU^+^ cells was comparable to FACS results.

### GFP uptake by macrophages infiltrating Matrigel™ plaque

**Figure 5:**
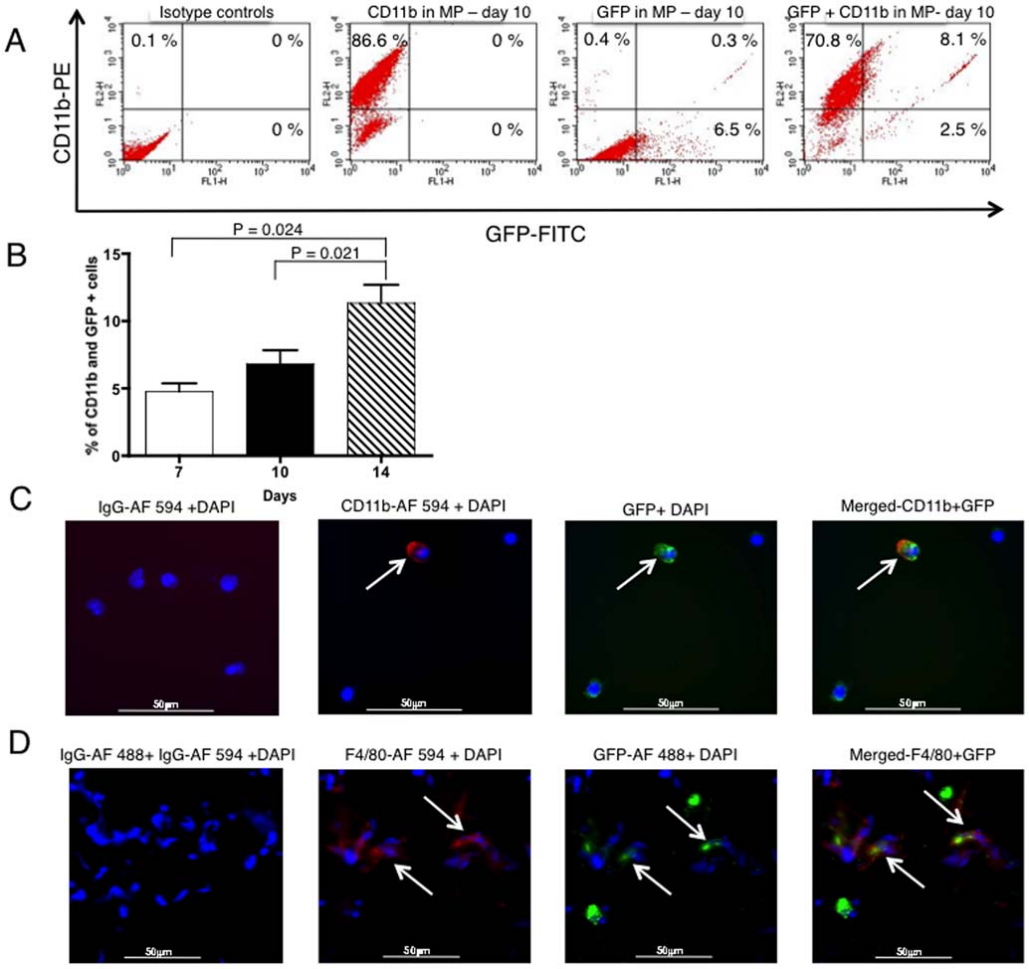
GFP uptake by local infiltrating Matrigel™ plaque macrophages from GFP-labeled hBMSCs. Representative flow cytometry dot plots showing 8.1% of CD11b-PE and GFP-FITC positive macrophages in MPs harvested ten days post-implantation (A), Bar graph showing the mean percentage of CD11b^+^ GFP^+^ cells in all MPs collected 7, 10 and 14 days after implantation of MPs containing 2×10^6^ xenogenic hBMSCs (B) Note gradual increase in the uptake of GFP by macrophages over time with statistically significant increase in the GFP uptake between day 7 and day 14 (p = 0.024) and day 10 and day 14 (p = 0.021). Immunofluorescent detection of GFP and CD11b with anti-mouse CD11b-Alexa Fluor 594 on cytospins of cells recovered from MPs ten days post-implantation (magnification 40X), (C). On the merged image, note cell only positive for GFP and a macrophage positive for GFP and CD11b-Alexa Fluor 594. Immunofluorescent detection of GFP and F4/80 with anti-GFP-Alexa 488 and anti-mouse F4/80-Alexa Fluor 594 on frozen sections of MPs ten days post-implantation (magnification 40X), (D). Cells positive for GFP and macrophage marker, F4/80, are indicated by arrows. Data are presented as a mean±1 SD from 12–18 plaques.

To assess the percentage of tissue macrophages taking up endogenous label, human BMSCs were transduced with a lentivirus encoding a GFP transgene. The FACS analysis of MP containing GFP- labeled hBMSCs revealed a trend of increasing frequency of CD11b^+^ GFP^+^ cells over time (4.5%±2.54 on day 7, 4.8%±2.17 on day 10 and 10.1%±3.5 on day 10) (Figure 5A and 5B). There was a statistically significant increase in the percentage of CD11b^+^ GFP^+^ cells found between day 7 and day 14 (p = 0.024 and day 10 and 14 (p = 0.021) (Figure 5B). The CD11b^+^ GFP^+^ cells were visualized on cytospins and frozen sections using fluorescence microscopy (Figure 5C and 5D). The percentage of CD11b^+^ GFP^+^ cells was comparable to flow cytometry results.

### Fe-Pro uptake by macrophages infiltrating Matrigel™ plaque

**Figure 6:**
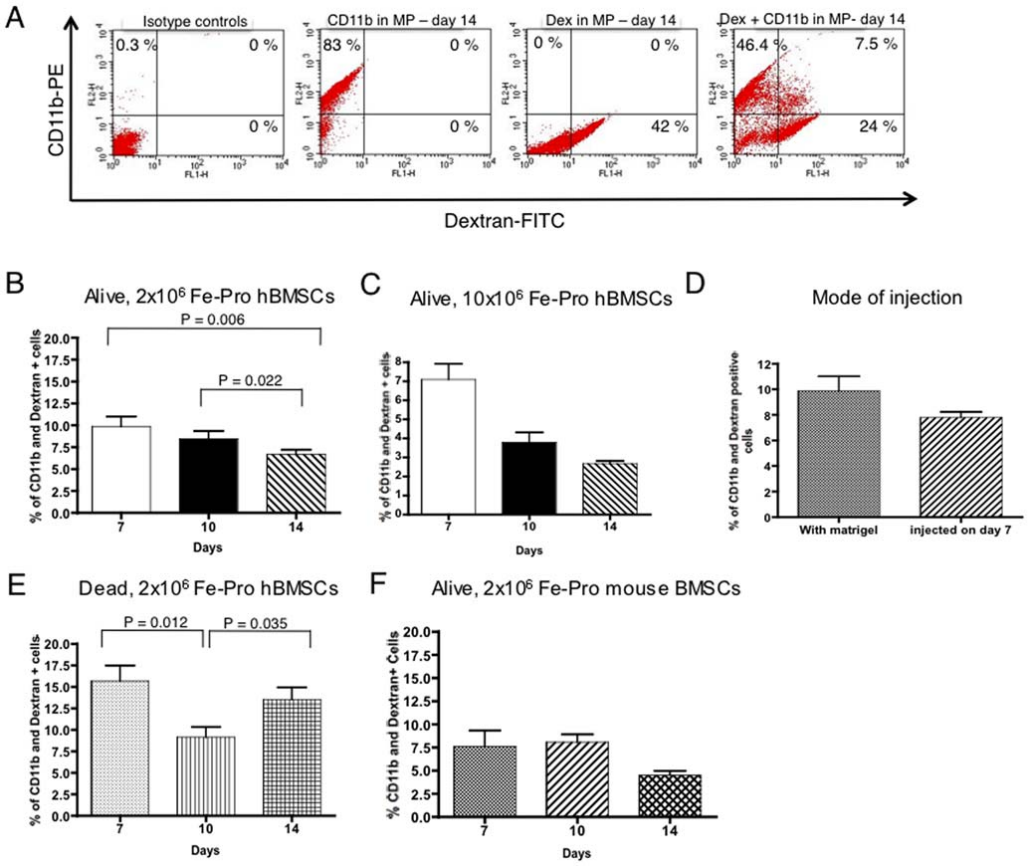
Fe-Pro uptake by local infiltrating Matrigel™ plaque macrophages from Fe-Pro-labeled BMSCs. Representative flow cytometry plots showing 7.5% of CD11b-PE and Dextran (Dex)-FITC positive macrophages in MPs harvested fourteen days post-implantation (A), Bar graph showing the mean percentage of CD11b^+^ Dex^+^ cells in all MPs collected 7, 10 and 14 days after implantation of MPs containing 2×10^6^ Fe-Pro labeled live xenogenic hBMSCs (B), 10×10^6^ Fe-Pro labeled live xenogenic hBMSCs (C), the mean percentage of CD11b^+^ Dex^+^ cells in all MPs containing 2×10^6^ Fe-Pro labeled hBMSCs and collected on day 7 after implantation as comparted to local injection of 2×10^6^ Fe-Pro labeled hBMSCs into already vascularized plaque and collecting it 7 days post-injection (D), bar graph showing the mean percentage of CD11b^+^ Dex^+^ cells in MPs collected 7, 10 and 14 days after implantation of MPs containing 2×10^6^ dead Fe-Pro labeled hBMSCs (E) and 2×10^6^ syngeneic, live Fe-Pro labeled mouse BMSCs (F). Note similar levels and a gradual decrease of Fe-Pro uptake by macrophages after implantation of live xenogenic Fe-Pro-labeled hBMSCs or syngeneic mouse Fe-Pro labeled BMSCs. The uptake of Fe-Pro by macrophages from dead Fe-Pro labeled hBMSCs was higher as compared to the Fe-Pro uptake from live Fe-Pro labeled hBMSCs.

The *in vivo* model of localized inflammation and angiogenesis associated with the MP perfusion assay was also used to determine the percentage of tissue macrophages migrating into the plaque and phagocytosing dextran coated iron oxide nanoparticles. Seven days after implantation of plaques containing two million live Fe-Pro labeled hBMSCs, 9.9%±4.13 of cells were found to be CD11b^+^dextran (Dex)^+^ (Figure 6A and 6B). Over time, there was a decreasing trend in the percentage of CD11b^+^Dex^+^ cells found on day 10 and 14 (8.5%±3.16 and 6.7%±1.91, respectively, Figure 6B). The decrease was statistically significant between days 7 and 14 (p = 0.006) and days 10 and 14 (p = 0.022) (Figure 6B). Implantation of MPs with a higher dose of live hBMSCs (10×10^6^ Fe-Pro labeled cells) resulted in similar trends, but with a slightly lower level of Fe-Pro uptake as compared with implantation of 2×10^6^ cells (Figure 6C). Flow cytometry analysis revealed 7.1%±1.67 of CD11b^+^ Dex^+^ cells on day 7, 3.8%±0.86 on day 10 and 2.7%±0.26 on day 14 post-implantation (Figure 6C). We also tested whether local injection into the already vascularized plaque of two million live Fe-Pro labeled hBMSCs on day 7 after implantation of the matrigel containing only growth factors, chemokines and LPS as opposed to implantation of cells and MP at the same time, would result in a different outcome. The analysis of the plaques containing locally injected cells showed similar level of uptake, of 7.8%±1.08 of CD11b^+^ Dex^+^ cells seven days after injection of cells as compared with 9.7%±4.13 of CD11b^+^ Dex^+^ cells found in plaques harvested seven days after cells were injected post MPs implantation (Figure 6D).

In the next experiment, we evaluated whether implanted dead Fe-Pro labeled hBMSCs would more readily transfer the label. Flow cytometry analysis of plaques implanted with 2 million dead Fe-Pro labeled hBMSCs resulted in higher uptake of the label when compared to the macrophage uptake of Fe-Pro from the plaques with live Fe-Pro labeled hBMSCs (Figure 6E). We observed 15.7%±4.33 of CD11b^+^ Dex^+^ cells on day 7, 9.1%±2.87 on day 10 and 13.6%±3.35 14 days following MPs implantation (Figure 6E). The statistically significant decrease in the percentage of CD11b^+^ Dex^+^ cells were found between day 7 and 10 (p = 0.012) and increase between day 10 and 14 (p = 0.035) (Figure 6E). When we compared the Fe-Pro uptake by macrophages from dead and live Fe-Pro labeled hBMSCs, the statistically significant increase in the uptake was observed on day 7 (p = 0.012) and day 14 (p<0.001).

**Figure 7:**
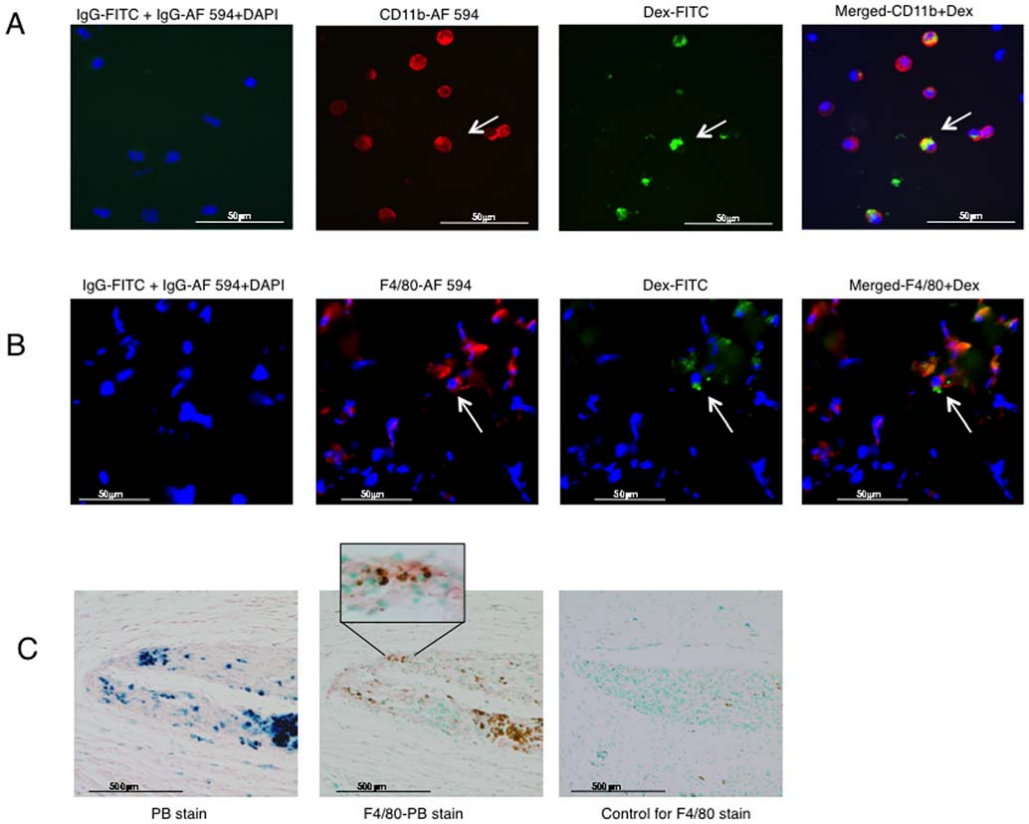
Immunofluorescent and Prussian Blue (PB) staining of Fe-Pro uptake by local infiltrating Matrigel™ plaque macrophages from Fe-Pro-labeled BMSCs. Representative immunofluorescent images showing detection of dextran (Dex) with anti-Dex-FITC, CD11b with anti-mouse CD11b-Alexa Fluor 594 on cytospins of cells recovered from MPs fourteen days post-implantation (magnification 40X), (A). On the merged image, note cells only positive for CD11b-Alexa Fluor 594 and macrophages positive for Dex-FITC and CD11b-Alexa Fluor 594. Those cells are local macrophages that took up Fe-Pro from Fe-Pro labeled hBMSCs. Immunofluorescent detection of Dex with anti-Dex-FITC, F4/80 with anti-mouse F4/80-Alexa Fluor 594 on frozen sections of MPs fourteen days post-implantation (magnification 40X), (B). Cells positive for macrophage marker, F4/80, as well as Dex are indicated by arrows. Immunohistochemical staining of MP sections showing colocalization of iron-positive cells (PB staining, blue or brown cells) and cells positive for macrophage marker, F4/80 (red cells) (C). Data are presented as a mean±1 SD from 12–18 plaques.

We next tested whether implantation of MPs with syngeneic mouse BMSCs would result in lower uptake of the Fe-Pro by tissue macrophages as seen in the case of BrdU uptake by macrophages from BrdU labeled, syngeneic mouse BMSCs. Flow cytometry revealed that seven days after implantation, MPs contained 7.6%±4.43 of CD11b^+^ Dex^+^ cells (Figure 6F). On day 10, we found 8.1%±1.89, while on day 14, 4.5%±1.39 of cells were CD11b^+^ Dex^+^ (Figure 6F). The CD11b^+^ Dex^+^ cells were visualized on cytospins and frozen sections by fluorescence microscopy (Figure 7A and 7B) as well as by immuno-histochemical staining (Figure 7C). The frequency of CD11b^+^ Dex^+^ as well as dextran and Prussian blue positive macrophages was comparable to FACS results.

**Figure 8:**
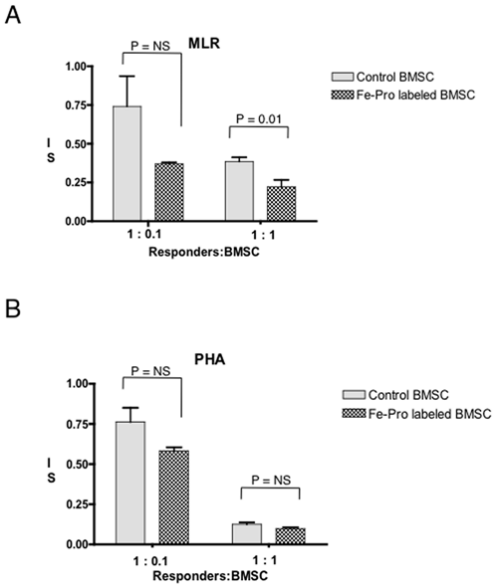
Mixed lymphocyte reaction (MLR) with Fe-Pro labeled hBMSCs. Bar graph showing the mean stimulation index (SI) for both, Fe-Pro labeled and unlabeled hBMSCs suppress T-cell proliferation in HLA mismatched MLR assay in response to alloreactive T-cells (A) and following nonspecific mitogen stimulation with phytohemagglutinin (PHA) (B). Control hBMSCs or Fe-Pro labeled hBMSCs were added at the ratios of 1∶1 or 0.1∶1 of non-irradiated PBMCs (responders) which proliferate in response to foreign histocompatibility antigens present on irradiated PBMCs (stimulators). Note, both the unlabeled control and Fe-Pro labeled hBMSCs suppressed MLR allo-reactivity in a dose-dependent manner. At the 1∶1 responder to hBMSCs ratio, the PBMC induced stimulation index was significantly lower with Fe-Pro labeled hBMSCs compared to control hBMSCs (p = 0.010), NS = not significant.

The lack of difference in the level of Fe-Pro uptake by tissue macrophages between xenogenic and syngeneic Fe-Pro labeled BMSCs (Figure 6B vs Figure 6F) prompted us to investigate the potential immuno-stimulatory properties of Fe-Pro in labeled cells. It is well established that BMSCs have the ability to suppress alloreactive T-cells *in vitro* in mixed lymphocyte reaction (MLR) assay [35]. Both the unlabeled control and Fe-Pro labeled hBMSCs suppressed MLR allo-reactivity in a dose-dependent manner (Figure 8). At the 1∶1 responder (non-irradiated peripheral blood mononuclear cells (PBMC) to hBMSCs ratio, the PBMC induced stimulation index was lower with Fe-Pro labeled hBMSCs compared to control unlabeled hBMSCs (22.0% vs 38.5%, p = 0.010) and there was a non-significant (p = 0.08) trend towards a lower stimulation index at the 1∶0.1 ratio (36.9% vs 74.1%, Figure 8A). There was no statistically significant difference in suppression of mitogen stimulated PBMC proliferation by Fe-Pro labeled BMSCs compared to unlabeled BMSCs (Figure 8B).

**Figure 9:**
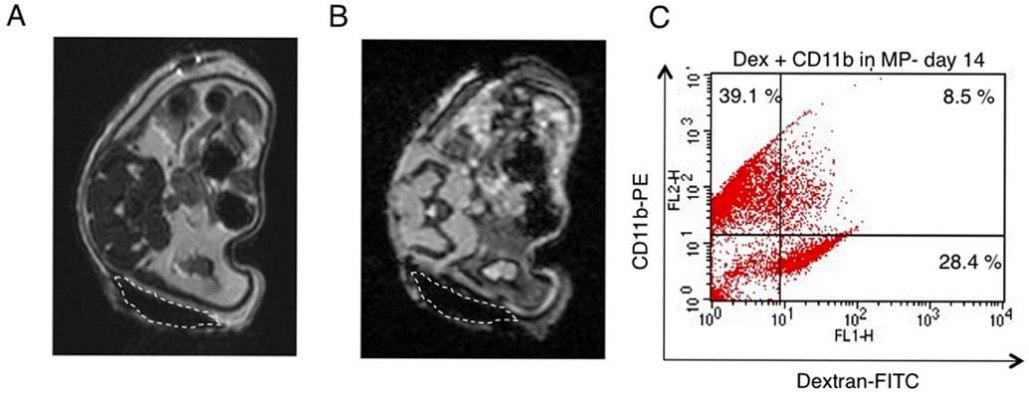
In vivo MRI T2 and T2* image of Matrigel™plaques (MPs) two weeks post-implantation. T2 weighted (A) and T2 * images (B) images of a representative MP contanining 2×10^6^ Fe-Pro labeled hBMSCs (indicated by white dashed line). Corresponding flow cytometry dot plot showing 8.5% of CD11b-PE and Dextran (Dex)-FITC positive macrophages in the imaged MPs (C).

MRI findings demonstrated unilateral hypointense regions corresponding to the area of MPs implanted with 2 million labeled hBMSCs (Figure 9A and 9B). Flow cytometry analysis of MPs imaged and harvested on day 14 post-implantation showed 8.5%±1.2 CD11b^+^ Dex^+^ macrophages (Figure 9C).

## Discussion

The major findings of the study are that the direct implantation of GFP, BrdU or SPION –labeled BMSCs into a localized area of inflammation can result in 5%–15% of the label being incorporated into host tissue macrophages. A mouse model of angiogenesis known as the Matrigel™ plaque perfusion assay was used and modified to simulate a controlled locally contained inflammatory environment where labeled cells would be locally transplanted into a site as a part of the cell therapy approach. The addition of growth factors such as vascular endothelial growth factor (VEGF-B) and angiopoietin-2 stimulated angiogenesis within the MPs (Figure 1). LPS and two potent stimulators of macrophages migration; chemokine MCP-1 and SDF-1 were also mixed within the MP prior to implantation to stimulate migration of macrophages and other cells into and out of the damaged and inflamed localized region. Flow cytometry analysis of harvested plaques revealed approximately 5% of BrdU^+^, 5%–10% GFP^+^ and 5%–15% Dex^+^ macrophages. These *in vivo* findings are consistent with our previously published *in vitro* study using a modified Boyden Chamber model of inflammation, where we observed approximately 10% BrdU from BrdU-labeled cells was transferred into macrophages and about 15% of macrophages were dextran positive and contained approximately10% of the total iron load from BMSCs [30]. To the best of our knowledge, this is the first *in vivo* study that provides the quantitative data on the transfer of exogenous or endogenous labels from BMSCs to host macrophages that are found in the inflamed tissues.

Recently, several published reports have indicated that intracellular labels from BMSCs or other stem cells can be transferred to host macrophages following direct transplantation of labeled cells. Two studies by Amsalem et al and Terrovitis et al used xenogeneic and syngeneic rat models of myocardial infarction where they injected iron oxide- and/or β-galactosidase- labeled rat or human BMSCs into the injured myocardium of immuno-competent rats [21], [22]. Using immuno-histochemical staining, the authors showed that three to four weeks after transplantation none of the labeled BMSCs could be detected with most of the Prussian blue positive cells for iron being infiltrating macrophages that participated in the rejection process. As a result, the hypointense region in the myocardium on MRI could represent a false positive for stem cells although the authors noted animals that received unlabeled stem cells also contained Prussian blue positive macrophages with the iron originating from local hemorrhage. Van de Bos et al reported that hemosiderin 5 weeks following myocardial reperfusion in the swine model resulted in a decrease in signal intensity on MRI that could mimic intracardiac implanted SPION labeled cells [36]. These results indicate that endogenous iron from hemorrhage can lead to Prussian blue positive macrophages within tissues. It is important to realize that with time the hypointense signal intensity area within the heart or other pathological tissue will decrease in areas as macrophages metabolize the iron oxide or migrate out of the tissue [37]–[39]. In other studies of direct transplantation of SPION labeled cells in mouse models of myocardial infarction, the transfer of the label to macrophages was reported to occur rarely [40]–[41]. In a severe skeletal muscle injury model, Winkler et al reported that local transplantation of SPION labeled autologous mesenchymal stem cells (MSCs) into the injured rat muscle resulted in a transfer of nanoparticles to ED-1 positive tissue macrophages based on histological analysis [42]. Other reports have indicated that ferumoxides can also be incorporated into the tissue macrophages from labeled neural progenitor cells following transplantation of labeled cells into the adult rat spinal cord as well as from ferumoxides labeled – pancreatic islet cells following their transplantation in mouse model of islet transplantation [28], [43]. The composition of the MPs contained 8.5% CD11b^+^Dex^+^ cells determined by FACS indicating that these labeled cells did not contribute significantly to the signal changes on MRI.

Burns et al [23] reported the transfer of BrdU or other thymidine analogs from labeled multipotent adult progenitor cells (MAPCs) to neurons or glia in the developing and adult brain. Furthermore, a recent study by Molcanyi et al [29] demonstrated in a fluid- percussion injury model, that GFP-transfected murine embryonic stem cells, once implanted into the injured rat brain, were phagocytosed by activated macrophages. Guzman et al showed 15% of local tissue macrophages were positive for BrdU following transplantation of BrdU tagged neural stem cells in a stroke model [44]. Collectively, all mentioned studies caution on the interpreting the results from pre-labeled transplanted cells to host cells, but none have quantified the frequency of the transfer of intracellular labels to host macrophages that phagocytosed the label or labeled cells.

The quantitative data presented in the current study together with our previously published *in vitro* study provides an estimate to aid in the interpretation of future transplantation studies. The results suggest that direct transplantation studies with cells labeled with BrdU, GFP or iron oxide nanoparticles should be rigorously validated by injections of unrelated live or dead cells and staining for markers expressed by local host cells such as tissue macrophages. This may be of particular importance in studies that involve direct injections of cells into highly inflamed or injured tissues such as myocardial infarction, or proliferative environments of central nervous system, where ≥90% of cells may die shortly after implantation [21], [23].

Our study showed that implantation of syngeneic BrdU-labeled BMSCs resulted in a lower uptake of BrdU by mouse macrophages compared to xenogenic hBMSCs. However, the uptake of SPION by macrophages was equivalent after implantation of syngeneic mouse Fe-Pro labeled BMSCs or xenogenic Fe-Pro-labeled hBMSCs. Since we used immuno-competent mice, this observation prompted us to test whether SPION labeling may predispose labeled cells to become more immunogenic and susceptible to an immune attack. The results of MLR assay revealed that the suppression of proliferation of alloreactive T-cells was surprisingly greater for Fe-Pro labeled hBMSCs when compared to the level of suppression achieved with unlabeled cells, indicating a possible decrease in immunoreactivity of labeled versus unlabeled cells. The lack of a difference in the level of SPION uptake from xenogenic Fe-Pro labeled hBMSCs compared to syngeneic mouse labeled BMSCs can be from greater immunosuppression of Fe-Pro labeled hBMSCs. In comparison, the implantation of xenogenic cells resulted in a lower immunoreactivity of the Fe-Pro labeled hBMSCs. Since clinical relevance of these data is not clear, additional *in vitro* and *in vivo* studies will be required to fully explain these results.

For this focal model of inflammation based on the MP perfusion assay, the dose of cells implanted into Matrigel™ plaques was based on our previously published *in vitro* Boyden Chamber model of inflammation [30]. Injection of higher doses such as ten million cells resulted in a similar level of label uptake by tissue macrophages compared to injecting two million cells. The implantation of dead Fe-Pro labeled hBMSCs as well as comparing the injection of labeled cells at the time of MPs implantation to direct injection of labeled cells once the plaque was vascularized, resulted in no difference in the level of Fe-Pro uptake.

In our previous study, we showed that less than 10% of the total iron from Fe-Pro labeled BMSCs was found in activated macrophages [30]. We concluded that the contribution of iron containing macrophages to the generation of hypointense regions observed on *in vivo* MRI was below the detection level. The results of this study are in accordance with that observation. MR imaging of MPs containing 2 million Fe-Pro labeled BMSCs showed no contribution of iron-containing tissue macrophages to the generation of signals on MRI. However, for studies on trans-differentiation, such as mesenchymal or hematopoetic stem cells differentiating to cells of other tissue like neurons or epithelial cells, interpretations based on the uptake of the label can be misleading especially since a very low percentage of cells are thought to change lineages and this may well be from the macrophage uptake of labels [45]–[46].

As in our previous report, the quantification of iron uptake by tissue macrophages was based on the flow cytometry analysis of cells stained with anti-dextran antibodies. We showed this technique to be highly reproducible, and a reliable method of determining the number of labeled BMSCs up to 4 days post-labeling. It is known that dextran may begin detaching from the iron oxide crystal as early as 24 hours after labeling depending on the size of the iron oxide nanoparticles and the type of cells labeled [47]. Furthermore, the behavior of SPION in labeled BMSCs is not known in the *in vivo* setting. In this study, we analyzed cells from MPs harvested one and two weeks post-implantation, therefore a limitation of this study could be a potential underestimation of the number of Fe-Pro labeled macrophages due to possible detachment of the dextran molecules from iron oxide crystal surface. Since we were unable to separate CD11b positive cells containing SPION from Fe-Pro labeled BMSCs it was not possible to determine if there were differences in the percentages of dextran positive from Prussian blue positive macrophages.

In conclusion, our data shows that direct implantation of BrdU, GFP or SPION labeled BMSCs into localized area of inflammation and angiogenesis can result in non-specific uptake of the label by infiltrating local tissue macrophages and lead to over - interpretation of the obtained results. Care should be taken to validate by histology of transplanted cells for macrophage markers, prior to drawing conclusions about the *in vivo* behavior of transplanted cells. Our study, which quantified the percentage of host bystander cells that up take the label from labeled BMSCs will aid in the interpretation of future transplantation studies.

## Materials and Methods

### Ethics statement

Animal experiments were performed according to a protocol approved by Clinical Center Animal Care and Use Committee (ACUC) of National Institutes of Health.

### Harvest and expansion of human and mouse bone marrow stromal cells (BMSCs)

Human and mouse 129/SvlmJ bone marrow stromal cells (BMSCs), a subset of which are multipotent skeletal stem cells, were obtained as described previously [1]. The methodological details are found in the Supporting Information Text S1.

### BMSCs transduction with lentivirus

Human BMSCs were transduced with a lentivirus encoding copeod green fluorescent protein (copGFP) (pSIH1-siLuc-copGFP lentivirus, System Biosciences, Mountain View, CA) by replacing the medium with fresh medium containing viral particles and incubating overnight at 37°C in a 95% air per 5% CO_2_ atmosphere. After overnight incubation, the media was replaced and the cells were incubated for 48 hours. Flow cytometry was used to evaluate the transduction efficiency of cells expressing the GFP transgene and transduced cells were sorted using the MoFlow cell sorter (Dako Cytomation, Fort Collins, CO).

### Labeling of BMSCs with BrdU

After reaching 60% confluence, human and mouse BMSCs were labeled with bromodeoxyuridine (BrdU) by adding 100 µg of BrdU (BD Biosciences, San Jose, CA) to culture flasks and incubating for 4 days. After labeling, cells were washed three times with sterile PBS, trypsinized and were then used in animal experiments. The labeling efficiency was evaluated by flow cytometry.

### Labeling of BMSCs with Fe-Pro

Ferumoxides (Fe, Feridex IV, Berlex Laboratories, Wayne, NJ) is a dextran coated SPIO nanoparticle approximately 120–150 nm in size and is provided at a total iron content of 11.2 mg/ml. Protamine sulfate (Pro, American Pharmaceuticals Partner, Schaumburg, IL), supplied at 10 mg/ml, was prepared as a fresh stock solution of 1 mg/mL in sterile distilled water immediately before labeling. Ferumoxides at a concentration of 100 µg/ml was added to a 50 ml conical tube containing serum-free RPMI 1640 (Biosource, Camarillo, CA) with 25 mM HEPES, MEM nonessential amino acids, sodium pyruvate, and L-glutamine. Protamine sulfate was added to the solution at 6 µg/ml and mixed for 2 minutes with intermittent hand shaking. Culture media was aspirated from the flasks containing human and mouse BMSCs and replaced with medium containing Fe-Pro complexes. After 2 hours of incubation at 37°C, an equal amount of relevant complete medium was added for a final concentration of Fe to pro, 50 µg/ml to 3 µg/mL, respectively. Cells were incubated overnight, approximately 16 hours, and washed three times with sterile PBS containing 10 U/mL heparin sulfate (American Pharmaceuticals Partner, Schaumburg, IL). Complete medium was added to each flask and labeled cells were kept in culture for 2 days to ensure all Fe-Pro complexes were endocytosed and the label was not attached to cell surface. In order to obtain dead cells, Fe-Pro labeled hBMSCs underwent 2–3 rounds of quick freeze/thaw cycles.

### Prussian Blue Staining and Fe-Pro Labeling Efficiency

To visualize the iron within Fe-Pro labeled cells, Prussian blue (PB) staining was performed. After 2 days post-labeling, BMSCs were trypsinized and transferred to cytospin slides. Cells were fixed with 4% glutaraldehyde, washed, and incubated for 30 minutes with 2% potassium ferric-ferrocyanide (Perl's reagent for staining, Sigma, St. Louis, MO) in 3.7% hydrochloric acid. Cells were washed again, counterstained with nuclear fast red and evaluated for iron staining using light microscopy (Axioplan Imaging II; Zeiss, Oberkochen, Germany) at X 40/0.75 objective lens and Axiovision 4.4 software (Zeiss, Oberkochen, Germany). The images were processed using Adobe Photoshop 7.0 (San Jose, CA). Fe-Pro labeling efficiency was determined by manual counting of PB stained and unstained cells using a Zeiss microscope (Axioplan Imaging II, Zeiss, Oberkochen, Germany) at X 100 magnification using an X 100/1.30 oil immersion objective lens. The percentage of labeled cells was determined from the average of 5 high-powered fields.

### Matrigel™ plaque perfusion assay

The Matrigel™ plaque perfusion assay was designed as previously described with modifications to simulate localized model of inflammation [31]–[33]. Briefly, 0.5 ml of Matrigel™ (ECM gel from Engelbreth-Holm-Swarm murine sarcoma, Sigma-Aldrich, St. Louis, MO) containing recombinant human protein VEGF-B_167_ and recombinant human angiopoietin-2, both at the dose of 500 ng/ml (both from R&D Systems, Minneapolis, MN), mouse recombinant SDF-1 and MCP-1 at the dose of 500 ng/ml (both from Peprotec, Rocky Hill, NJ), and lipopolisacharide (LPS) at the dose of 100 ng/ml was mixed with 2×10^6^ or 10×10^6^ human or mouse BMSCs labeled with GFP, BrdU or SPIO nanoparticles and incubated overnight at 37°C in a 95% air per 5% CO_2_ atmosphere. After overnight incubation, solidified Matrigel™ plaques (MPs) were injected or transplanted subcutaneously into both shaved and butadiene/alcohol prepped flanks of 6-week-old 129/SvlmJ mice (Charles River Laboratories, Inc., Wilmington, MA) under isoflurane anesthetized (2.5–3% isoflurane with an oxygen flow rate of 2.5 liters/min), 6–9 mice per group and time point. As controls, 4 mice in each group were injected with Matrigel™and growth factors alone. As an alternative experiment to implantation of Matrigel™ containing labeled cells, 6 mice were injected with Matrigel™ and growth factors only, and left for 7 days to allow the neovasculature to develop. At day seven, 2×10^6^ human SPION labeled BMSCs were locally injected into the plaque and left for an additional 7 days before removal.

On day 7, 10 and 14, the mice were euthanized and the skin of the mouse was pulled back to expose MPs. Plugs were then resected from surrounding connective tissue, placed into tubes containing dispase (BD Biosciences, San Jose, CA), snap-frozen for fluorescence microscopy, or fixed in 3.7% formaldehyde, embedded in paraffin and sectioned for immunohistochemistry staining. MPs were incubated with dispase for 2 hours at 37°C, then homogenized and centrifuged at low speed for 10 minutes, washed several times in PBS, resuspended in fresh RPMI medium and recovered cells were counted using a hemocytometer.

### Flow Cytometry Analysis (FACS)

Cells recovered from the plaques were fixed and permeabilized with BD Cytofix/Cytoperm Plus™ (with GolgiStop™containing monensin) kit (BD Biosciences, San Jose, CA) according to manufacturer's instructions. Cells were stained for dextran, GFP and CD11b using anti-dextran IgG_1_-FITC (Stem Cell Technologies Inc., Vancouver, Canada), anti-GFP-FITC (Millipore, Billerica, MA) and anti- CD11b IgG_2a_-PE (BD Biosciences, San Jose, CA) antibodies. The control experiments included unstained cells and cells stained with isotype controls for each of the antibodies. BrdU was detected using a FITC-BrdU Flow Kit (BD Biosciences, San Jose, CA) according to manufacturer's instructions.

In order to assess the cell content of the recovered plaques, representative MPs were stained for mouse CD45 (leukocyte common antigen), CD3 (lymphocytes), CD31 (endothelial cells) and human CD29 to identify BMSCs. The following antibodies were used: anti-human CD29- IgG_2a_-PE, anti –mouse CD31- IgG_2a_-PE, anti-mouse CD45 - IgG_1_-FITC, anti- mouse CD-3 IgG_1_-FITC (all from BD Biosciences, San Jose, CA) antibodies. Cells were also stained with propidium iodide (PI). Stained cells were analyzed by flow cytometry (FACS Calibur, Becton Dickinson, Franklin Lakes, NJ). A total of 15,000 cells were analyzed at the FL1, FL2 and FL3 detectors and the results were presented as the mean percentage of CD11b^+^ Dex^+^, CD11b^+^ GFP^+^ or CD11b^+^ BrdU^+^ cells from each recovered MP.

### Fluorescence Microscopy and Immunohistochemistry Analysis

For fluorescence microscopy and immunohistochemical analysis MPs were recovered and stained according to standard procedures. The materials and methods are described in detail in Supporting Information Text S1.

### Mixed Lymphocyte reaction (MLR) assay

The ability of BMSCs to suppress alloreactive T-cells has traditionally been assessed *in vitro* using a mixed lymphocyte reaction (MLR) assay [35]. Therefore, we compared the immunosuppressive properties of iron oxide labeled- hBMSC to control hBMSCs using a conventional 5-day MLR assay. Peripheral blood from two healthy, HLA-mismatched donors was collected via apheresis. Ficoll-separated peripheral blood mononuclear cells (PBMCs) were plated in a 96 well plate at 100,000 responder cells per well. Responder cells were either a) co-cultured with 2500 cGy irradiated stimulator PBMCs at a 1∶1 or b) stimulated with the mitogen phytohemagglutinin (PHA). Control hBMSCs or Fe-Pro labeled hBMSCs were added at the following responder-BMSCs ratios: 1∶0.1 or 1∶1. Culture plates were incubated for 5 days in a humidified 5% CO2 incubator at 37°C. On day 4 of the MLR, 1 µCi of ^3^H-thymidine (^3^H-TdR) was added to each well for 20 hours with T-cell proliferation measured using a liquid scintillation counter. The data are presented as stimulation index values calculated by using the following formula: proliferation of responder PBMCs stimulated by irradiated PBMCs (stimulators) or PHA and incubated with control or Fe-Pro labeled hBMSCs/proliferation of PBMCs stimulated by PBMCs or PHA alone. The experiments were performed at least three times for each variable described.

### Magnetic Resonance Imaging

Four 6-week-old 129/SvlmJ mice were implanted with MP containing 2×10^6^ Fe-Pro labeled BMSCs or unlabeled cells and MRI was performed on day 14-post implantation. The methodological details are found in the Supporting Information Text S1.

### Statistics

The experiments were performed at least three times for each variable described. Data are expressed as a mean±SD. The statistically significant differences were detected using the Student *t* test for unpaired data (2-tail) with significant p value<0.05 (Prism 4 Macintosh; GraphPad Software, Inc., San Diego, CA).

## Supporting Information

Text S1 (0.05 MB DOC)
