# Supplementary material for: In Vivo Transfer of Intracellular Labels from Locally Implanted Bone Marrow Stromal Cells to Resident Tissue Macrophages
Source: PLoS One. 2009 Aug 21;4(8):e6712. doi: 10.1371/journal.pone.0006712 (PMC2726631; doi:10.1371/journal.pone.0006712)
Supplement: Text S1 — (0.05 MB DOC) [file pone.0006712.s001.doc]

*Supplementary Methods*

**In Vivo Transfer of Intracellular Labels from Locally Implanted Bone Marrow Stromal Cells to Resident Tissue Macrophages.**

Edyta Pawelczyk, PhD^1^, Elaine K. Jordan, DVM^1^, Arun Balakumaran, MD, PhD ^2^, Aneeka Chaudhry, BS^1^, Nicole Gormley, MD^3^, Melissa Smith, BS^1^, Bobbi K. Lewis, BA^1^, Richard Childs, MD^3^, Pamela G. Robey, PhD^2^, Joseph A Frank, MD, MS^1,4^

^1^Radiology and Imaging Sciences, Clinical Center, National Institutes of Health, Bethesda, Maryland, USA, ^2^ National Institute of Dental and Craniofacial Research, National Institutes of Health, Bethesda, Maryland, USA, ^3^National Heart, Lung and Blood Institute, National Institutes of Health, Bethesda, Maryland, USA, ^4^National Institute of Biomedical Imaging and Bioengineering, National Institutes of Health, Bethesda, Maryland, USA

**Supplemental Methods**

# Harvest and expansion of human bone marrow stromal cells (BMSCs)

Fragments of bone with marrow were obtained from volunteers undergoing bone marrow biopsy under IRB approved procedures, in accordance with NIH regulations governing the use of human subjects. Fragments of trabecular bone and marrow were scraped gently with a steel blade into cold modified Minimum Essential Medium [alpha] MEM, (Life Technologies, Grand Island, NY), and pipetted repeatedly. The released marrow cells were passed consecutively through 16- and 20-gauge needles and filtered through a 70-μm pore size nylon cell strainer (Becton Dickinson, Franklin Lakes, NJ) to remove cell aggregates. Bone marrow single cell suspensions were plated at high cell density of 1.0×10^7^ nucleated cells per 75-cm^2^ flask (Becton Dickinson, Lincoln Park, NJ). Growth medium included [alpha]-MEM, 2 mM l-glutamine, 100 U/ml penicillin, 100 μg/ml streptomycin sulfate (Biofluids, Rockville, MD), and 20% lot-selected fetal bovine serum (Equitech-Bio, Kerkville, TX). Cells were cultured at 37°C in a 95% air per 5% CO_2_ atmosphere. For generating large numbers of BMSCs, high-density cultures were prepared in triplicate; the media was replaced on day 6 or 7. The cultures first passage was on day 12 with two consecutive applications of 1x trypsin-EDTA (Life Technologies, Gaithersburg, MD) for 5–10 min each at room temperature. The cells harvested from each 75-cm^2^ flask were counted separately using a Coulter Counter (Coulter Electronics, Inc, Hialeah, FL) and plated into a separate 175-cm^2^ flask (Becton Dickinson, Lincoln Park, NJ). The subsequent passages were performed at 4 to 7 day intervals. BMSCs were used at passages 3 to 5 for this study.

Mouse BMSCs were isolated from the femurs of 6-week-old 129/SvlmJ mice. Bones were removed aseptically, the marrow cells flushed with cold modified Minimum Essential Medium [alpha] MEM, (Life Technologies, Grand Island, NY) and combined. Cell suspension was pipetted repeadly, passed through 16- and 20-gauge needles and filtered through a 70-μm pore size nylon cell strainer (Becton Dickinson, Franklin Lakes, NJ) to yield a single cell suspension. Nucleated cells were counted via hemocytometer and seeded at cell density of 5.0×10^7^ cells per 75-cm^2^ flask (Becton Dickinson, Lincoln Park, NJ) in a medium composed of [alpha]-MEM, 2 mM l-glutamine, 100 U/ml penicillin, 100 μg/ml streptomycin sulfate (Biofluids, Rockville, MD), and 20% fetal bovine serum (Equitech-Bio, Kerkville, TX). Cells were cultured at 37°C in a 95% air per 5% CO_2_ atmosphere. After one day of incubation at 37°C the medium was replaced and changed three times a week for two weeks. At that time cultures became 70 % confluent. Cells were passed by washing with Hank’s balanced salt solution followed by two treatments with 1x trypsin-EDTA (Life Technologies, Gaithersburg, MD) for 5–10 min each at room temperature. Harvested cells were counted and plated in a 175-cm^2^ flask (Becton Dickinson, Lincoln Park, NJ). Mouse BMSCs were used at passage 3 for this study.

Since mouse BMSCs preparations are known for high levels of macrophage contamination, we magnetically depleted CD11b positive cells, before mouse BMSCs were used in animal experiments. Briefly, a trypsinized suspension of mouse BMSCs was counted and magnetically labeled with CD11b MicroBeads according to the manufacturer’s instructions (Miltenyi Biotec, Auburn, CA). Labeled cells were loaded onto MACS® columns (Miltenyi Biotec, Auburn, CA) and placed in the magnetic field. The CD11b positive cells were retained on the column while pure BMSC were eluted as the positively selected cell fraction. Cells were counted with a hemocytometer and the purity of the mouse BMSCs was confirmed by flow cytometry.

**Fluorescence Microscopy and Immunohistochemistry Analysis**

For fluorescence microscopy, cells recovered from the plaques were permeabilized and stained for BrdU, dextran, GFP, CD11b and F4/80 using the FITC-BrdU Flow Kit (BD Biosciences, San Jose, CA), anti-dextran IgG_1_-FITC (Stem Cell Technologies Inc., Vancouver, Canada), rat anti- mouse CD11b, rat anti mouse-F4/80 (both from AbD Serotec, Raleigh, NC) and rabbit-anti-GFP (Millipore, Billerica, MA) followed by secondary goat anti-rat IgG-Alexa Fluor 594 and rat anti-rabbit IgG-Alexa Fluor 488 (both from Invitrogen, Carlsbad, CA), respectively. Stained cells were transferred to cytospin slides, cover-slipped, mounted with VECTASHIELD mounting medium with 1.5 μg/ml DAPI (Vector Laboratories, Burlingame, CA) and analyzed by fluorescence microscopy (Axioplan Imaging II; Zeiss, Oberkochen, Germany) at X 40/0.75 objective lens and Axiovision 4.4 software (Zeiss, Oberkochen, Germany). The images were processed using Adobe Photoshop 7.0 (San Jose, CA). Cryostat sections (6-8 μm) were fixed in acetone and stained according to the same protocol as cells stained in suspension. For immunohistochemistry, representative MPs were fixed in 3.7% formaldehyde, embedded in paraffin and sectioned. Deparaffinization and rehydration of the 8-μm paraffin sections of MPs was followed by Prussian blue staining by 5-10 minute incubation with 2% potassium ferric-ferrocyanide (Perl’s reagent for staining, Sigma, St. Louis, MO) in 3.7% hydrochloric acid. After incubation, washing of sections was followed by antigen retrieval with 10 mM sodium citrate buffer (pH 6.0, 5 min in 700-W microwave) and cooling for 30 min at room temperature. Endogenous peroxidase activity was minimized by incubation in 3% H_2_O_2_ solution for 15 min, followed by incubation with blocking serum (provided in Vectastain® Elite ABC kit, Vector Laboratories, Inc, Burlingame, CA) in PBS, for 1 hour at room temperature. Incubation of sections with primary antibody for mouse F4/80 (AbD Serotec, Raleigh, NC) was performed overnight at 4C. This incubation step was omitted in negative control sections. Sections were subsequently stained with biotinylated secondary antibodies followed by formation of the avidin-biotin-peroxidase complex (Vectastain® Elite ABC kit, Vector Laboratories, Inc, Burlingame, CA) according to manufacturer’s instructions, followed by a 5-10 min-treatment with Vector™ NovaRED Substrate or DAB (both from Vector Laboratories, Inc, Burlingame, CA) for visualization of peroxidase activity at the antigen sites. Couterstaining of sections with Vector® Methyl Green (Vector Laboratories, Inc, Burlingame, CA), dehydration in graded alcohols and xylene, and mounting with Vectashield mounting medium (Vector Laboratories, Inc, Burlingame, CA) followed thereafter. Representative MP sections underwent hematoxylin and eosin (H& E) staining using standard methods.

**Magnetic Resonance Imaging**

In vivo MRI studies were performed on a 3T Achieva whole-body scanner (Philips Medical System, The Netherlands) using a dedicated 4 cm mouse solenoid RF-coil (Philips Research Laboratories, Hamburg, Germany) according to approved animal care and use committee protocol at our institution. Animals were anesthetized with 1.5 ~ 2% isofluorane and 100% oxygen delivered through a nosecone. Physiological monitoring was performed with SAII MRI compatible unit (Small Animal Instruments Inc., Stony Brook, NY). The MR pulse sequences were as follows: T2-weighted (T2w) turbo spin echo (TSE) sequence, TR/TE = 3200/60 ms, turbo spin echo factor 12, number of average (NAV) 8, field of view (FOV) 50 mm, slice thickness 0.5 mm, matrix 224×256, reconstructed resolution 100×100 µm, slice number 25. And a T2* multi echo gradient sequence (T2*w), TR/effective TE = 4560/28 ms, 15 echos, flip angle 30°, NAV 2, FOV 50 mm, slice thickness 0.5 mm, matrix 176×256, reconstructed resolution 200×200 µm. MRIs were analyzed for signal intensity changes and T2* values, MPs were removed to assess the cell content according to protocol described above.
